# Inhibition of eNOS by L-NAME resulting in rat hind limb developmental defects through PFKFB3 mediated angiogenetic pathway

**DOI:** 10.1038/s41598-020-74011-1

**Published:** 2020-10-07

**Authors:** Ziqiang Wu, Huan Yao, Huan Xu, Yang Wang, Wangming Hu, Guanhua Lou, Lingling Zhang, Cong Huang, Cen Jiang, Shiyi Zhou, Yaping Shi, Xiongbing Chen, Lan Yang, Yiming Xu, Yong Wang

**Affiliations:** 1 Chengdu University of Traditional Chinese Medicine, College of Basic Medicine, Chengdu, Sichuan China; 2 Chengdu University of Traditional Chinese Medicine, College Pharmacy, Chengdu, China; 3 School of Basic Medical Sciences, Guangzhou Medical University, Guangzhou, China

**Keywords:** Developmental biology, Drug discovery, Molecular biology, Stem cells, Medical research, Molecular medicine

## Abstract

l-arginine/NOS/NO signaling pathway plays a critical role in controlling variety of vascular diseases. However, whether NOS inhibition by L-NAME suppresses late embryonic development is undefined. The aim of this study is to determine whether NOS inhibition by L-NAME is critical for late embryonic rat hind limb development. The pregnant rat at E13.5 administrated L-NAME by consecutive intraperitoneal injection. The embryos been harvested from E16.5 to E 20.5. Hematoxylin and Eosin Staining, Immunofluorescence and Immunohistochemistry performed to determine hind limb Vasculogenesis, HUVEC culture, Adenoviral PFKFB3 infection, Real time PCR and western blot were performed to determine whether l-arginine/NOS/NO pathway controlling late embryonic hind limb development through PFKFB3 mediated angiogenetic pathway. NOS inhibition by L-NAME resulting in late embryonic hind limb developmental defects characterized by severe hemorrhage. The in vivo studies showed that NOS inhibition strongly suppressed hind limb angiogenetic remodeling by impairing differentiation of endothelial cells and smooth muscle cells, and extracellular matrix synthesis. For underlie mechanism, our studies indicated that L-NAME treatment dramatically suppresses PFKFB3 expression in hematopoietic progenitor cells, tubulogenetic endothelial cells and smooth muscle cells. Knockdown of PFKFB3 dramatically inhibits the expression of angiogenetic genes, as well as tubulogenesis and extracellular matrix related genes. Taken together, our data in this study demonstrated that l-arginine-eNOS-NO pathway is important for rat hind limb development during late embryonic stage. This could be both a useful animal model and a promising therapeutic treatment for defects of late embryonic developmental hind limbs.

## Introduction

Nitric Oxide (NO) plays a critical role in controlling variety of biological processes, including inhibition DNA synthase, mitogenesis and cell proliferation^1–4^. Endogenous NO is synthesized from l-arginine by Nitric Oxide Synthase (NOS), and there exists three distinct NOS isoforms: nNOS, iNOS, eNOS^5, 6^. Dysfunction of NO signaling pathway has been associated with pulmonary vascular disease^7^, pulmonary arterial hypertension^8^, atherosclerosis^9^, vascular inflammatory disease^10^, diabetes^11^, neurodegeneration disease^12^ and cancers^13^.

As a powerful vasodilator, NO is particularly critical in regulating vascular disease. Vascular endothelial NO has been reported to be a vital factor to protect damages of vessels from risk factors, including cigarettes smoke, high blood pressure, high glucose or high lipids^7^. Vascular endothelial NO suppresses CD11/CD18 to regulate leukocyte adhesion which is essential for onset of atherosclerosis development^6^. NO regulates vasculogenesis during embryo development stage^14,15^ and induces endothelial cell migration through activation of PI3K/Akt signaling pathway^16^. NO inhibits vascular smooth muscle cell proliferation^17^ and NO promotes extracellular matrix (ECM) production^18^. Inhibition of NO generation in vivo results in impaired vascular permeability induced by VEGF^19^. No inhibition results rats hind limb disruption^20^.

eNOS is the predominant NOS isoform in vascular system and account for most of vascular NO production^1^. eNOS-knockout male mice exhibits premature death and age-related cardiac dysfunction phenotype^21^. Pregnant eNOS knockout dams shown fetal growth restriction which characterized by vascular dysfunction and altered placental nutrient transportation^22^. eNOS inhibition caused malformation in rat fetus^23^. However, the underlie mechanism for rat late embryonic development defects is largely unknown.

NG-Nitro- l-Arginine Methyl Ester (L-NAME), a non-selective NO synthase inhibitor^24^, is widely used to inhibit nitric oxide synthase activity both in vivo and in vitro^25^. It is difficulty to monitor abnormalities during early embryonic development stage, but multiple instruments and experiment testing methods can be used to determine developmental defects during late embryonic development stage. This should benefit for reducing incidence of congenital malformation diseases. Here, we report that L-NAME inhibits NOS during late embryonic stage, which most critical organs have been formed, resulting in hind limb developmental defects. Moreover, we tried to define whether L-NAME treatment induced NOS inhibition dramatically suppresses vasculogenesis through PFKFB3 mediated vascular endothelial cell glycolysis.

## Materials and methods

### Methods

#### Ethical approval

The use of rat approved by the Experimental Animal Ethics Committee of Chengdu University of Traditional Chinese Medicine in accordance with NIH guidelines. Ethical approval number: 2019-04.

### Animal treatment

Rat estrous cycle monitored by vaginal smear observation before breeding, the day by noon when vaginal plug observed defined as embryonic day 0.5 (E0.5). L-NAME purchased from sigma (Sigma Prod. No. N5751). The pregnant rat at E13.5 administrated L-NAME (50 mg/kg) by consecutive intraperitoneal injection^26^. Sacrificed the rats in an airtight tank filled with carbon dioxide, check the heart beating before collected the embryos. Placed embryos in the airtight tank filled with carbon dioxide again. Harvested the hind limb after carefully check Breathing and heartbeat. The embryos been harvested and undergoing whole mount observation and the hind limb undergoing paraffin-embedded process.

### Hematoxylin and Eosin (HE) Stain and Immunohistochemistry (IHC) and immunofluorescence staining (IF)

The hind lambs from rat embryos were fixed with 4% paraformaldehyde overnight at 4 °C and undergoing paraffin embedded, 5-μm thickness of slides were collected. Hematoxylin/eosin (HE) staining performed as previously described^27^. For IHC staining, the deparaffinized slides were treated with citric acid and antigenic unmasked at 98 °C for 5–10 min, incubated with primary antibodies overnight at 4 °C, followed by incubation with biotinylated secondary antibody at room temperature for 1 h (Vector Laboratories, 1:200), and ABC solution (Vector Laboratories, Burlingame, CA) for 30 min at room temperature. Expression of the targets visualized after DAB solution added. Antibodys used in this study: CD31 (Biocare; mouse, 1:200); SM α-actin (Sigma, mouse, 1:200); MHC (Biomedical Technologies Inc, rabbit 1:100) and PFKFB3 (Proteintech; rabbit, 1:100). For IF staining, after antigen retrieval process, block antigen with goat serum, incubated with primary antibody, including CD31, smooth muscle α-actin α-actin and PFKFB3 antibody, following incubated with Alexa 594 or 488-confugated secondary antibodies, nuclei visualized with 4′, 6′-diamidino-2-phenylindole (DAPI) staining, images were captured using confocal microscopy (LS510, Zeiss).

### Cell culture

Human umbilical vein/vascular endothelial (HUVEC, ATCCPCS-100-013) was purchased from ATCC and cultured in with vascular cell basal medium (ATCC, PCS-100-030) supplemented with endothelial Cell Growth Kit (ATCC, PCS-10-040).

### Adenoviral PFKFB3 generation and HUVEC infection

Adenovirus encoding PFKFB3 generated as described previously^28^. This virus contains another independent cytomegalovirus promoter-driven transcription cassette for green fluorescent protein (GFP) in addition of PFKFB3. The infection efficiency can directly be monitored by visualization of the expression of GFP.

### Quantitative real time PCR analysis

The real time PCR performed as our previous publications^27,29^. Total RNA from HUVEC was extracted using TRIzol reagent. 500 ng RNA were using as template for reverse transcription with random hexamer primers using iScript cDNA synthesis kit. Real time PCR performed duplicated on ABI real time PCR system with gene specific primers that listed in Table 1. Relative gene expression level was analysis using the 2^−∆∆ct^ method against β-actin.

**Table 1:** List of primer sequences used for quantitative RT-PCR.

Gene name	Species	Sequence
PFKP	Human	F: 5ʹ-GCATGGGTATCTACGTGGGG-3ʹ
	Human	R: 5ʹ-CTCTGCGATGTTTGAGCCTC-3ʹ
PFKFB3	Human	F: 5ʹ-CTCGCATCAACAGCTTTGAGG-3ʹ
	Human	R: 5ʹ-TCAGTGTTTCCTGGAGGAGTC-3ʹ
GLUT1	Human	F: 5ʹ-GGCCAAGAGTGTGCTAAAGAA-3ʹ
	Human	R: 5ʹ-ACAGCGTTGATGCCAGACAG-3ʹ
HK1	Human	F: 5ʹ-GCTCTCCGATGAAACTCTCATAG-3ʹ
	Human	R: 5ʹ-GGACCTTACGAATGTTGGCAA-3ʹ
GPI	Human	F: 5ʹ-CAAGGACCGCTTCAACCACTT-3ʹ
	Human	R: 5ʹ-CCAGGATGGGTGTGTTTGACC-3ʹ
PGK1	Human	F: 5ʹ-TGGACGTTAAAGGGAAGCGG-3ʹ
	Human	R: 5ʹ-GCTCATAAGGACTACCGACTTGG-3ʹ
LDHA	Human	F: 5ʹ-ATGGCAACTCTAAAGGATCAGC-3ʹ
	Human	R: 5ʹ-CCAACCCCAACAACTGTAATCT-3ʹ
LDHB	Human	F: 5ʹ-TGGTATGGCGTGTGCTATCAG-3ʹ
	Human	R: 5ʹ-TTGGCGGTCACAGAATAATCTTT-3ʹ
PDK1	Human	F: 5ʹ-CTGTGATACGGATCAGAAACCG-3ʹ
	Human	R: 5ʹ-TCCACCAAACAATAAAGAGTGCT-3ʹ
ALDOA	Human	F: 5ʹ-ATGCCCTACCAATATCCAGCA-3ʹ
	Human	R: 5ʹ-GCTCCCAGTGGACTCATCTG-3ʹ
TIE2	Human	F: 5ʹ-TTGAAGTGGAGAGAAGGTCTG-3ʹ
	Human	R: 5ʹ-GTTGACTCTAGCTCGGACCAC-3ʹ
VEGFR1	Human	F: 5ʹ-TCTCACACATCGACAAACCAATACA-3ʹ
	Human	R: 5ʹ-GGTAGCAGTACAATTGAGGACAAGA-3ʹ
VEGFR2	Human	F: 5ʹ-GCAGGGGACAGAGGGACTTG-3ʹ
	Human	R: 5ʹ-GAGGCCATCGCTGCACTCA-3ʹ
VEGFR3	Human	F: 5ʹ-GACAGCTACAAGTACGAGCATCTG-3ʹ
	Human	R: 5ʹ-CGTTCTTGCAGTCGAGCAGAA-3ʹ
VEGF121	Human	F: 5ʹ-CCCTGATGAGATCGAGTACATCTT-3ʹ
	Human	R: 5ʹ-GCCTCGGCTTGTCACATTTT-3ʹ
VEGF165	Human	F: 5ʹ-CCCTGATGAGATCGAGTACATCTT-3ʹ
	Human	R: 5ʹ-AGCAAGGCCCACAGGGATTT-3ʹ
VEGF185	Human	F: 5ʹ-CCCTGATGAGATCGAGTACATCTT-3ʹ
	Human	R: 5ʹ-AACGCTCCAGGACTTATACCG-3ʹ
ANGPT1	Human	F: 5ʹ-AACATGGGCAATGTGCCTACACTT-3ʹ
	Human	R: 5ʹ-CATTCTGCTGTATCTGGGCCATCT-3ʹ
ANGPT2	Human	F: 5ʹ-CAGATTTTGGACCAGACCAGTGA-3ʹ
	Human	R: 5ʹ-TCAATGATGGAATTTTGCTTGGA-3ʹ
CD148	Human	F: 5ʹ-AGTACACACGGCCCAGCAAT-3ʹ
	Human	R: 5ʹ-GAGGCGTCATCAAAGTTCTGC-3ʹ
NRP1	Human	F: 5ʹ-CAGAAAAGCCCACGGTCAT-3ʹ
	Human	R: 5ʹ-CAGCCAAATTCACAGTTAAAACC-3ʹ
NRP2	Human	F: 5ʹ-AAGTCTCCTACAGCCTAAACGG-3ʹ
	Human	R: 5ʹ-GATGTCAGGGGTGTCATAGTGC-3ʹ
RHOA	Human	F: 5ʹ-GAAGAGGCTGGACTCGGATT-3ʹ
	Human	R: 5ʹ-AGCAAGCATGTCTTTCCACA-3ʹ
F3	Human	F: 5ʹ-CACTACAAATACTGTGGCAG-3ʹ
	Human	R: 5ʹ-TCCAATCTCCTGACTTAGTG-3ʹ
EGFL7	Human	F: 5ʹ-TGGATGAATGCAGTGCTAGG-3ʹ
	Human	R: 5ʹ-CCTTGGGCACACAGAGTGTA-3ʹ
Notch1	Human	F: 5ʹ-GTTCTTGCAGGGGGTGC-3ʹ
	Human	R: 5ʹ-GGTGAGACCTGCCTGAATG-3ʹ
Notch2	Human	F: 5ʹ-CAACTCGATGAGTGTGCGTC-3ʹ
	Human	R: 5ʹ-ATGCCCTGGATGGAAAATGGA-3ʹ
Notch3	Human	F: 5ʹ-ATGCAGGATAGCAAGGAGGA-3ʹ
	Human	R: 5ʹ-AAGTGGTCCAACAGCAGCTT-3ʹ
Notch4	Human	F: 5ʹ-TGTGAACGTGATGTCAACGAG-3ʹ
	Human	R: 5ʹ-ACAGTCTGGGCCTATGAAACC-3ʹ
Jagged1	Human	F: 5ʹ-CAGGACCTGGTTAACGGATTT-3ʹ
	Human	R: 5ʹ-GCCTCACATTTGCATC-3ʹ
Jagged2	Human	F: 5ʹ-AGGTGGAGACGGTTGTTACG-3ʹ
	Human	R: 5ʹ-TTGCACTGGTAGAGCACGTC-3ʹ
DII4	Human	F: 5ʹ-GCCTATCTGTCTTTCGGGCT-3ʹ
	Human	R: 5ʹ-ATTGTGGGGGATGCATTCGT-3ʹ
Fibronectin	Human	F: 5ʹ-CCGCCGAATGTAGGACAAGA-3ʹ
	Human	R: 5ʹ-TGCCAACAGGATGACATGAAA-3ʹ
Integrin	Human	F: 5ʹ-TGGGCTACCGGGCAGAG-3ʹ
	Human	R: 5ʹ-CAGCATTAACAGCAACAATCCG-3ʹ
CD31	Human	F: 5ʹ-TGTATTTCAAGACCTCTGTGCACTT-3ʹ
	Human	R: 5ʹ-TTAGCCTGAGGAATTGCTGTGTT-3ʹ
GJC1	Human	F: 5ʹ-AGCTGTAGGAGGAGAATCCATC-3ʹ
	Human	R: 5ʹ-TGCAAACGCATCATAACAGACA-3ʹ
EFNB2	Human	F: 5ʹ-TTCGACAACAAGTCCCTTTG-3ʹ
	Human	R: 5ʹ-GATGTTGTTCCCCGAATGTC-3ʹ
VE-CAD	Human	F: 5ʹ-GAGCCGCCGCCGCAGGAAG-3ʹ
	Human	R: 5ʹ-CGTGAGCATCCAGCAGTGGTAGC-3ʹ
VWF	Human	F: 5ʹ-GTCGAGCTGCACAGTGACATG-3ʹ
	Human	R: 5ʹ-GCACCATAAACGTTGACTTCCA-3ʹ
VCAM1	Human	F: 5ʹ-TCAGATTGGAGACTCAGTCATGT-3ʹ
	Human	R: 5ʹ-ACTCCTCACCTTCCCGCTC-3ʹ
ICAM1	Human	F: 5ʹ-GGCCGGCCAGCTTATACAC-3ʹ
	Human	R: 5ʹ-TAGACACTTGAGCTCGGGCA-3ʹ
iNOS	Human	F: 5ʹ-GCAGAATGTGACCATCATGG-3ʹ
	Human	R: 5ʹ-ACA ACCTTGGTGTTGAAGGC-3ʹ
eNOS	Human	F: 5ʹ-TGATGGCGAAGCGAGTGAAG-3ʹ
	Human	R: 5ʹ-ACTCATCCATACACAGGACCC-3ʹ
nNOS	Human	F: 5ʹ-CAGCCCAATGTCATTTCTGTT-3ʹ
	Human	R: 5ʹ-GATCACGGGCGGCTTACT-3

### Protein preparation and Western blotting

The western blotting experiment performed as our previous publications^29,30^. Protein from cells were extracted using RAPI buffer containing protease inhibitor. Protein concentration was quantified using BCA assay and separated with SDS-PAGE gel. The antibodies used in this study were β-actin (Cell Signaling Technology; mouse, 1:3000); PFKFB3 (Proteintech; rabbit, 1:1000). Images captured by using ImageQuan LAS4000 Imaging Station (GE) and the densities of bands were quantified using the ImageQuant TL software (GE).

### Statistics

The animal numbers required for statistical significance determined by power analysis. Each groups were determined in a randomized manner. The data analyzed using GraphPad Prism Software by one-way ANOVA with Tukey’s post-hoc test or Student’s t-test to evaluate two-tailed levels of significance. Data presented as mean ± SEM, P ≤ 0.05 was consider statistically significant.

## Results

### NOSs inhibition leads to rat late embryonic hind limb developmental defects

**Figure 1:**
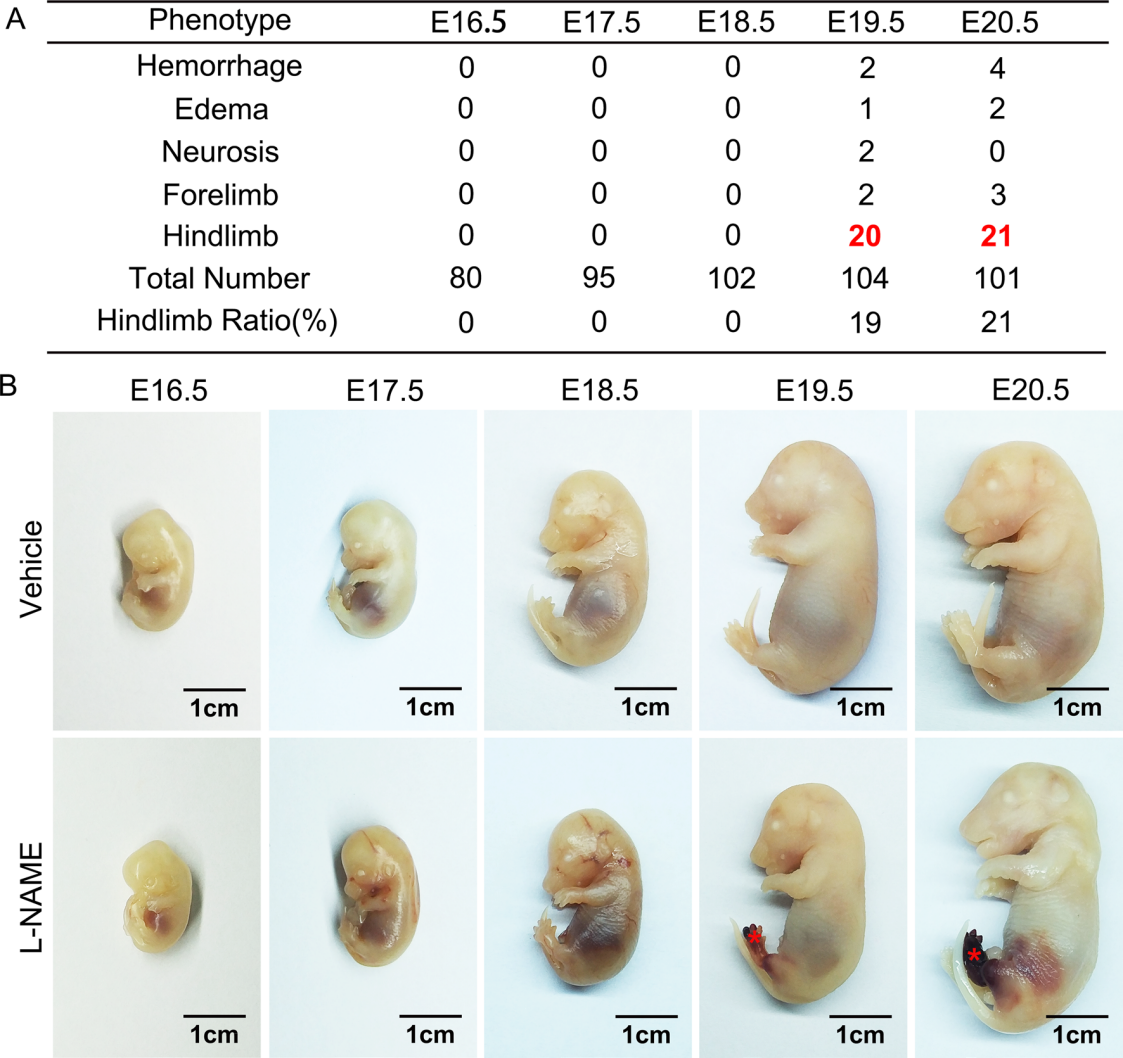
NOSs inhibition leads to late embryonic hind limb developmental defect. (**A**) Pregnant rats were consecutive administration L-NAME that started at embryonic day 13.5 (E13.5) by peritoneal injection, the embryos were harvest at different time point and multiple phenotypes were check. L-NAME administration induced embryonic hind limb developmental defects, which account for 20%. Approximately 5% embryos displayed fore limb hemorrhage. In addition, no more than 2%, shown cerebral defect, or embryonic lethality. (**B**) Whole mount images to show hind limb developmental defect after inhibition of NO production. Images obtained from Nikon SMZ745 microscope, Scale bar: 1 cm.

NO is a strong vasodilator and regulates blood vessel relaxation, thus NO is involved in hypertension and atherosclerosis^31,32^. Althrough the functions of NO have been extensive studied in adult stage, the role of NO in late embryonic hind limb development and underlie mechanism is largely unknown. To determine whether NO is essential for late embryonic development, we reduced NO synthesis by consecutive intraperitoneal injection of L-NAME, an unselected NOS inhibitor, to pregnant rat starting from embryonic day 13.5 (E13.5). No obvious difference observed between L-NAME and correspondent control group from E16.5 to E17.5. However, NO deficient embryos exhibited conspicuous abnormal phenotype at E19.5 characterized by hind limb hemorrhage, edema, cerebral development defect, lethality etc. The majority phenotype is the hind limb defects exhibited by hemorrhage, small size, shape deformed and edema, which account for more than 20%. Approximately 5% embryos displayed fore limb hemorrhage. Only a small number, no more than 2%, shown cerebral defect, or embryonic lethality (Fig. 1A,B). These results demonstrated deficient of NOS induced by L-NAME plays a pivotal role in regulating rat late embryonic hind limb development, the dramatically hemorrhage phenotype may be associated with cardiovascular defects.

### NOSs inhibition impairs rat late embryonic hind limb vasculogenesis

**Figure 2:**
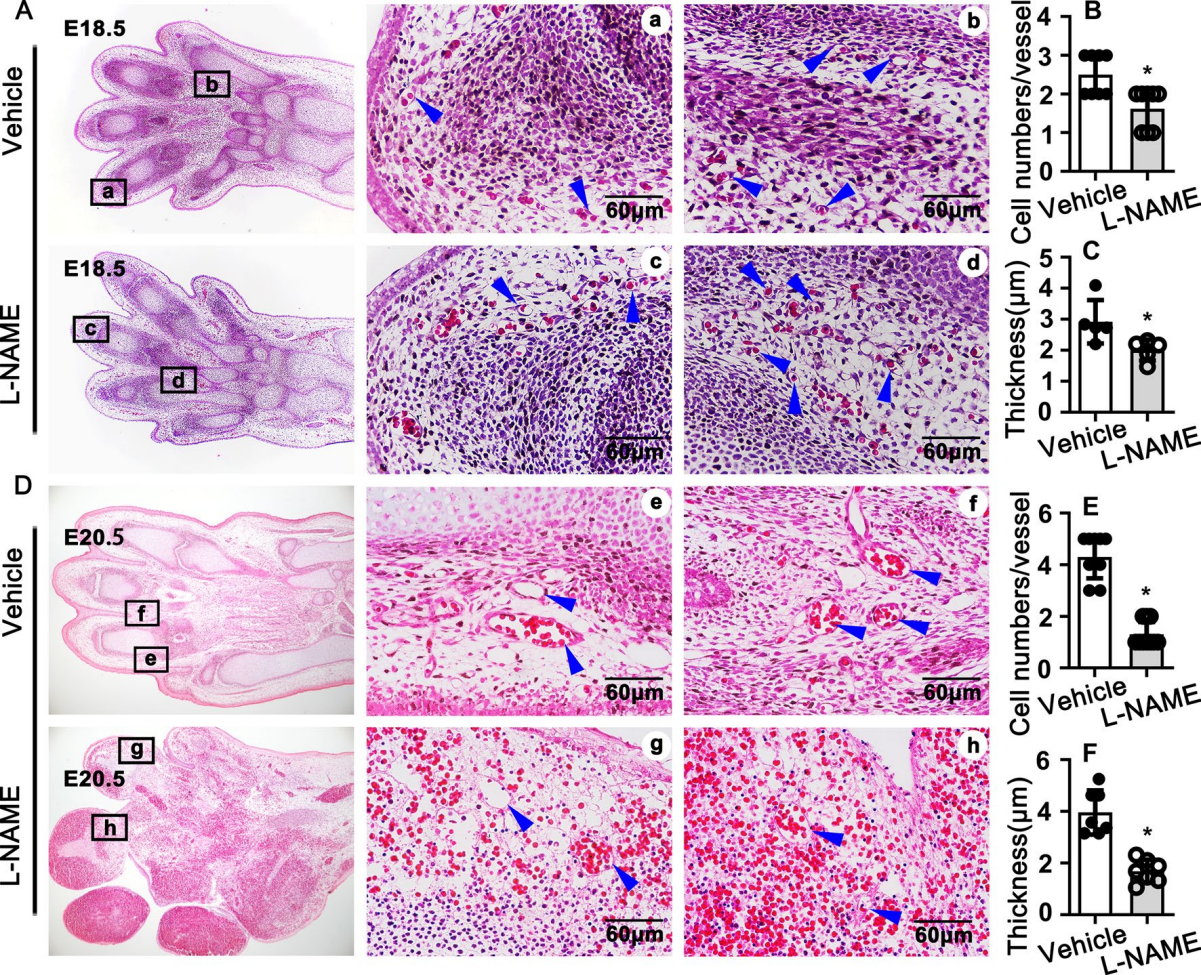
Suppression of NO production impairs hind limb angiogenesis. (**A**) Representative Images of HE staining from E18.5 to exhibit the vessel structure within hind limb, the whole images exhibited in left and corresponding areas exhibited in middle and right. Quantification analysis of endothelial cell numbers for each vessel and the thickness of vessel displayed in (**B**) (n = 8 mice per group, unpaired t-test) and (**C**) (n = 5 mice per group, unpaired *t*-test). (**D**) Representative Images of H&E staining from E20.5 and the cell numbers and vessel thickness quantified in (**E**) (n = 9 mice per group, unpaired *t*-test) and (**F**) (n = 7 mice per group, unpaired *t*-test). Scale bar: 60 μm. Data are presented as means ± SEM, *P < 0.05.

To define whether the hind limb developmental defect is due to an impairment of vascular development, we performed HE stain to investigate Vasculogenesis process during embryonic development. Both control and NOS deficient embryos showed that nonucleated hematopoietic precursor cells retained in undifferentiated mesoderm cells at E16.5 (S Figure 1A). The nucleus of hematopoietic precursor displayed which was close to the cell membranes at E17.5. The undifferentiated mesoderm cells changed its regular circle shape to spindle structure, and embraced the nucleated hematopoietic cells, which contribute to the development of the pretubular structure at E17.5 (S Figure 1C). No difference of the cell numbers based on nuclear hematoxylin for vessels between NOSs inhibition group and control groups (S Figure 1B,D). Our data indicates that impairment of NOS has no effect on both the differentiation of hematopoietic precursor cells and initiation the development of mesoderm cells to form the prevascular tubular structures. Although at E18.5, the NOS deficient embryos exhibited normal shape, and normal size of hind limb, the cell number of each vessels and the thickness of each vessel were dramatically decreased (Fig. 2A–C). More serious phenotype displayed at E19.5, which characterized hemorrhage leakage from vessels, as well as decreased cell numbers and vascular wall thickness (S Figure 1F–H). The data mentioned above further demonstrated that deficiency of NOSs did not influents the differentiation of nonucleated hematopoietic precursor cells to nucleated blood cells. At E20.5, the control embryos exhibited the five-finger shape, the well-organized vessels and maturated nonucleated blood cells within the vessels. However, the NOSs deficient embryos presented abnormal shape, especially the lack of vascular network in hind limb characterized by blood leakage, impairment of the vessels structures, the missing of mesoderm cells, abnormalized of five fingers, as well as edema (Fig. 2D). In addition, both the cell numbers of each vessels and the thickness of the vessel wall were significantly deceased (Fig. 2E,F). Our data suggested that the NO signal pathway plays a pivotal role during rat late embryonic hind limb Vasculogenesis development.

### NOSs inhibition impairs rat late embryonic hind limb vascular endothelial cells development

Endothelial cells are critical for embryonic Vasculogenesis. After the initial vessels formed via Vasculogenesis during embryonic development, the circulatory system is developed and matured via angiogenesis. One of the best-known angiogenetic mechanism is called sprouting angiogenesis. The tubulogenesis phase involved in tip cells migration and stalk cells proliferation. The angiogenic remodeling phase involved in stabilization of endothelium by recruitment of mesenchymal cells that differentiated into pericytyes and smooth muscle cells^33–35^.

Endothelial cells are essential for vasculogenesis characterized by proliferation of stalk cells and migration of tip cells^36^ . Since L-NAME is a Non-selective NO synthase inhibitor, and there are three isoforms has been identified, including nNOS, iNOS, eNOS. We first determine which isoform dominant expressed in endothelial cells. The expression level of eNOS is much higher than that of iNOS or nNOS (S Figure 2A). L-NAME treatment dramatically inhibited eNOS expression, while not influenced the expression of iNOS and nNOS (S Figure 2B). Those data demonstrated L-NAME inhibits NO production from l-arginine through eNOS dependent pathway in endothelial cells.

**Figure 3:**
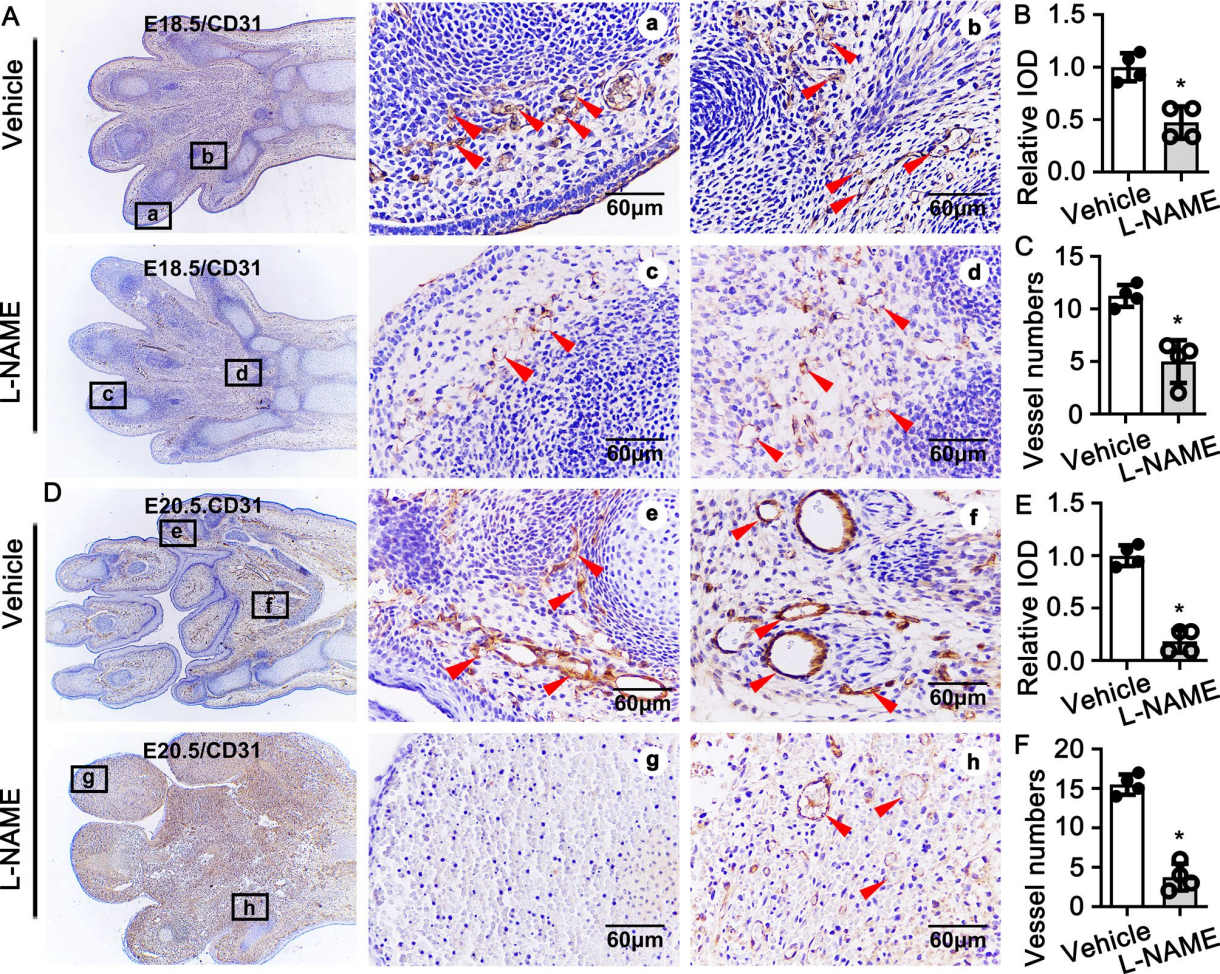
Suppression of No production suppresses vascular endothelial cells development and maturation. (**A**) IHC staining against CD31 antibody to visualize the vascular endothelial cells development at E18.5. Relative expression of CD31 according to IOD and small vessel numbers quantified in (**B**) (n = 4 mice per group, unpaired *t*-test) and (**C**) (n = 4 mice per group, unpaired t-test). The vessel development at E20.5 displayed at (**D**,**E**) (n = 4 mice per group, unpaired t-test) and (**F**) (n = 4 mice per group, unpaired *t*-test). Scale bar: 60 μm. Data presented as means ± SEM, *P < 0.05.

We next sought to determine whether eNOS inhibition suppresses endothelial functions. Since HUVEC is a maturated cell type, the role of eNOS inhibition on development of late embryonic endothelial cell is largely unknown. We sought to determine whether NO is essential for initial the differentiation and maturation of endothelial cell. The endothelial-like cells were circular around hematopoietic cells. However, there was no difference of cell numbers of each vessel between the control group and NO deficient at both E16.5 and E17.5, as well as the integral optical density (IOD) which was determined by immunohistochemistry staining against CD31 to visualization the vascular endothelial cells (S Figure 3A–F). Our data demonstrated that NO was not essential for initiation the differentiation of late embryonic endothelial cells. At E18.5, there were tubular structures appeared within the control group. However, the IOD and the vessels numbers dramatically decreased within eNOS inhibition group (Fig. 3A–C). The similar results exhibited at E19.5, and serious hemorrhage displayed at this stage (S Figure3G–I). It is difficult to detect small tubular structure at E20.5 due to serious hemorrhage, and the vessel numbers of the big vessels obviously decreased, as well as the IOD (Fig. 3D–F). The data descripted above indicates that eNOS inhibition did not impair the initiation of endothelial cell differentiation, as well as the migration of endothelial cells, but impaired endothelial cells maturation.

### NOSs inhibition impairs rat late embryonic hind limb vascular smooth muscle recruitment and development

**Figure 4:**
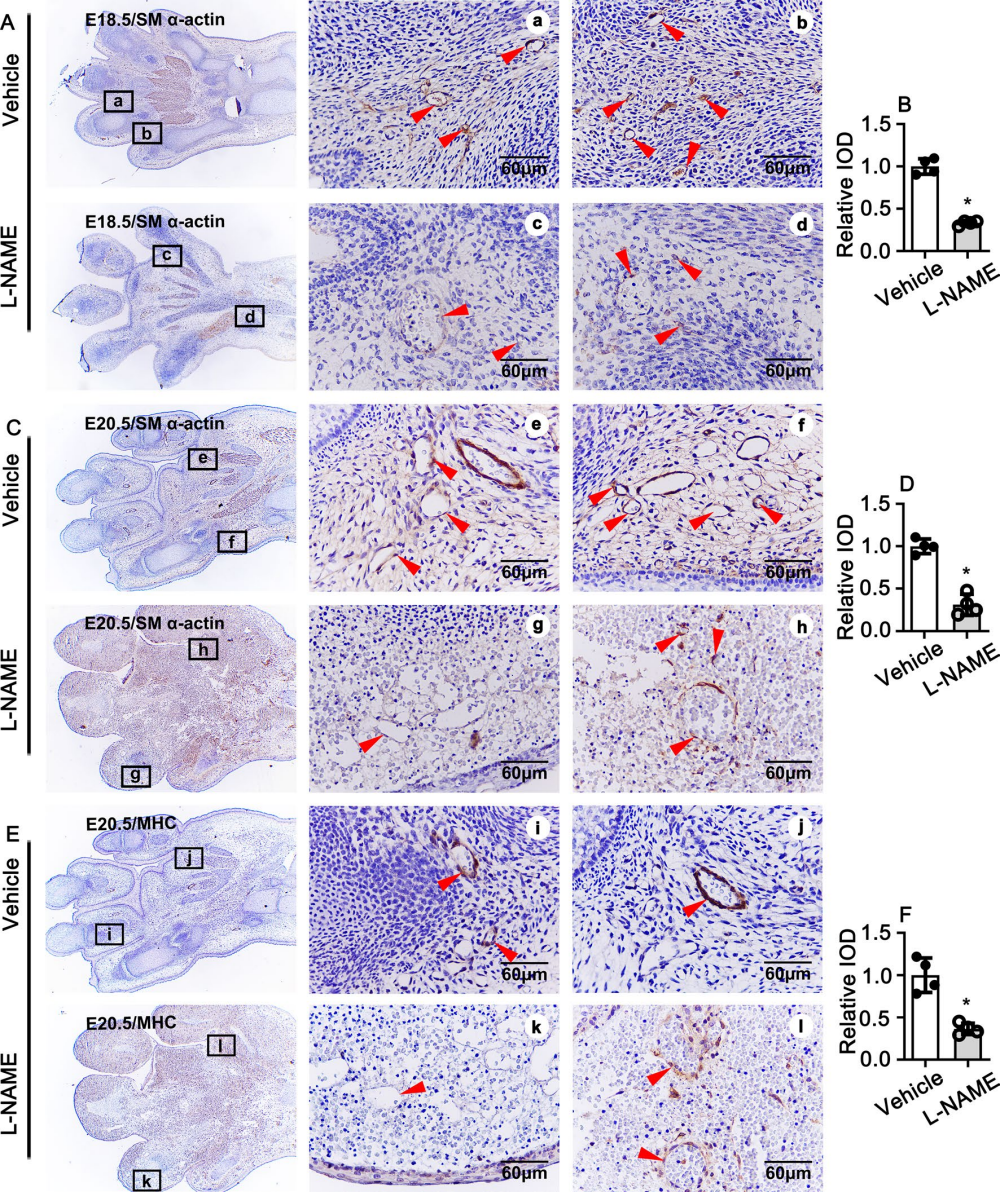
Suppression of NO production impairs smooth muscle cell development and recruitment. (**A**) IHC staining against smooth muscle α-actin (SM α-actin) to visualize the vascular smooth muscle cell development at E18.5. (**B**) Relative expression of SM α-actin in A quantified which based on the IOD (n = 4 mice per group, unpaired *t*-test). IHC staining against SM α-actin at E20.5 and quantification displayed in (**C**,**D**) (n = 4 mice per group, unpaired *t*-test). IHC staining against MHC at E20.5 and quantification displayed in (**E**,**F**) (n = 4 mice per group, unpaired *t*-test). Scale bar: 60 μm. Data presented as means ± SEM, * *P* < 0.05.

**Figure 5:**
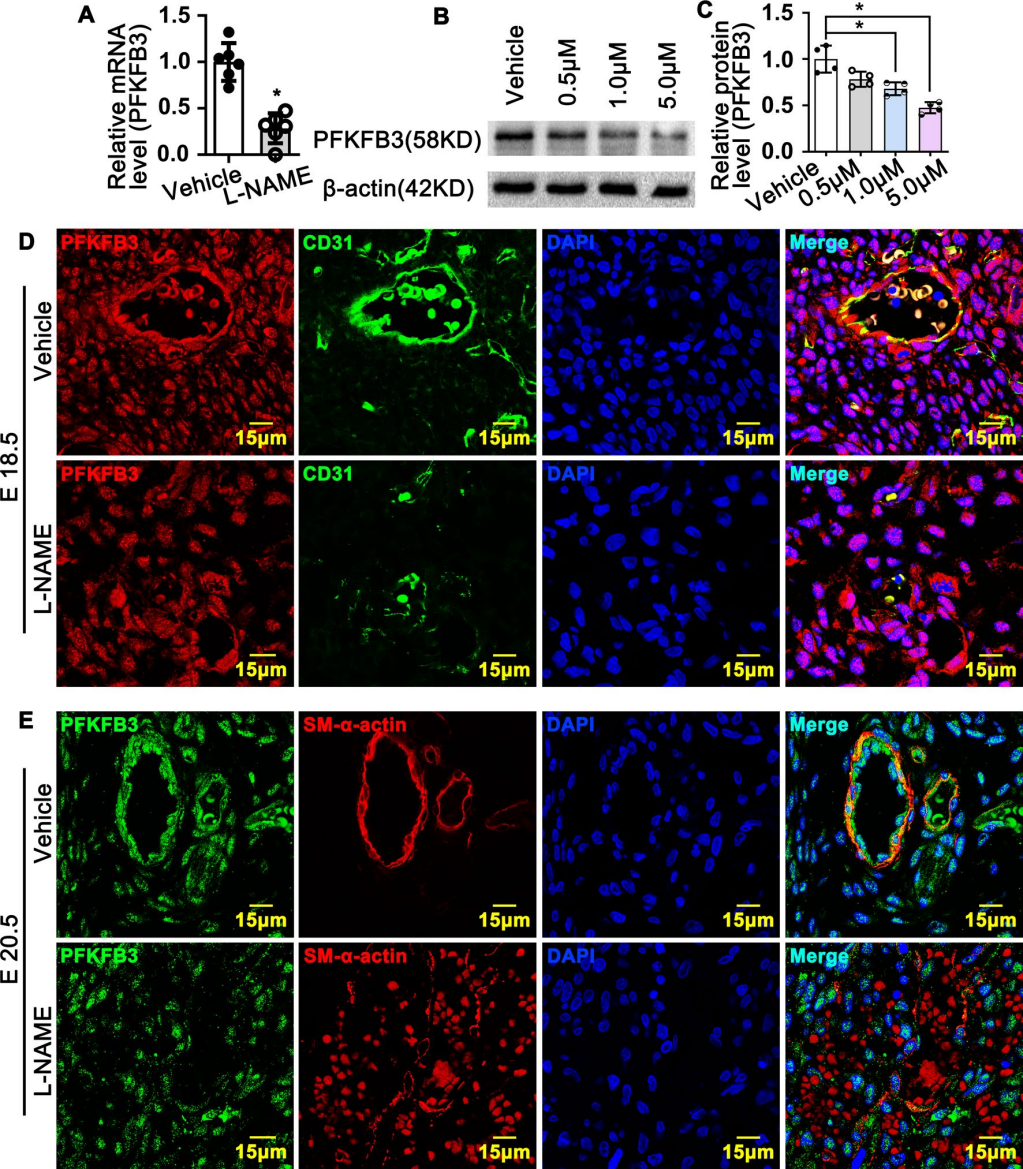
Suppression of NO production decreases the expression of PFKFB3 in vivo and in vitro. (**A**) HUVEC treated with L-NAME (1 μM) for 30 h and Real Time PCR performed to detect the expression of PFKFB3 (n = 6 independent experiments, paired *t*-test). (**B**) HUVEC treated with different dose of L-NAME (0.5 μM, 1.0 μM and 5.0 μM) for 48 h and western blot performed to investigate PFKFB3 protein level. PFKFB3 protein levels in (**B**) quantified in (**C**) (n = 4 independent experiments, paired *t*-test). (**D**) IF staining against with PFKFB3 and CD31 antibodies at E18.5, (**E**) IF staining against with PFKFB3 and CD31 antibodies at E20.5. Scale bar: 15 μm. Data presented as means ± SEM, *P < 0.05.

Endothelium is a single cell layer, which covers within the vessels and protects our body from damaged stress. Following the proliferation of stalk cells and migration of tip cells, the recruitment of smooth muscle cells is particularly important for stabilization of the now formed tubular structures. We performed IHC staining against smooth muscle marker genes, including smooth muscle α-actin (SM α-actin), myosin heavy chain (MHC), to detect whether blocking NO production can impair smooth muscle cells development and recruitment during rat late embryonic stage. At E16.5, the expression of SM α-actin cannot detect in both control group and L-NAME treated group (Data not shown). At E17.5, SM α-actin expression observed in large vessels, as well as within the new formed tubular vessels. However, no significant difference exhibited between the control group and L-NAME treated group based on the quantification of integral optical density (IOD) (S Figure 4A,B). Inhibition of eNOS by L-NAME has no effect on the vessel number, but dramatically decrease the expression of SM α-actin at E18.5 (Fig. 4A,B). This phenomenon further confirmed at E19.5, which characterized by remarkable decreased the vessel numbers, the thickness of the vessels and enlargement of the lumen of vessels, at the same time, serious hemorrhage observed (S Figure 4C,D). The similar phenotype exhibited at E20.5 (Fig. 4C,D). The impairment of smooth muscle development detected by IHC experiment against myosin heavy chain antibody. Positive expression signal of MHC only detected within large vessels at E16.5 (Data not shown). Comparable expression of MHC between the control and L-NAME treated group detected in both large vessels and new formed tubular vessels at E17.5 (Data not shown). Seminary results exhibited at E18.5, no different of the vessel numbers, the thick of the vessels, the lumen of the vessels, as well as the cell number for each vessel displayed between the control and L-NAME group (S Figure 5A,B). Nevertheless, observable decreased expression of MHC exhibited within L-NAME treated group; at the same time, the decreased of the numbers of vessels, the thickness of the vessels also observed both in E19.5 and E20.5 (S Figure 5C,D; Fig. 4E,F). Those results indicated that inhibition the eNOS by L-NAME has no affection on the initiation of smooth muscle cells development and recruitment, but it is essential for vascular maturation during late embryonic stage.

### NOSs inhibition impairs rat late embryonic hind limb extracellular matrix deposition

The formation of a new basement membrane contains extracellular matrix is critical for stabilization of new vessels^37–39^. The adhesion of endothelial cell to extracellular matrix is essential for endothelial cell proliferation, migration, survival, morphogenesis and blood vessel stabilization, which are also essential for neovascularization^38^.

**Figure 6:**
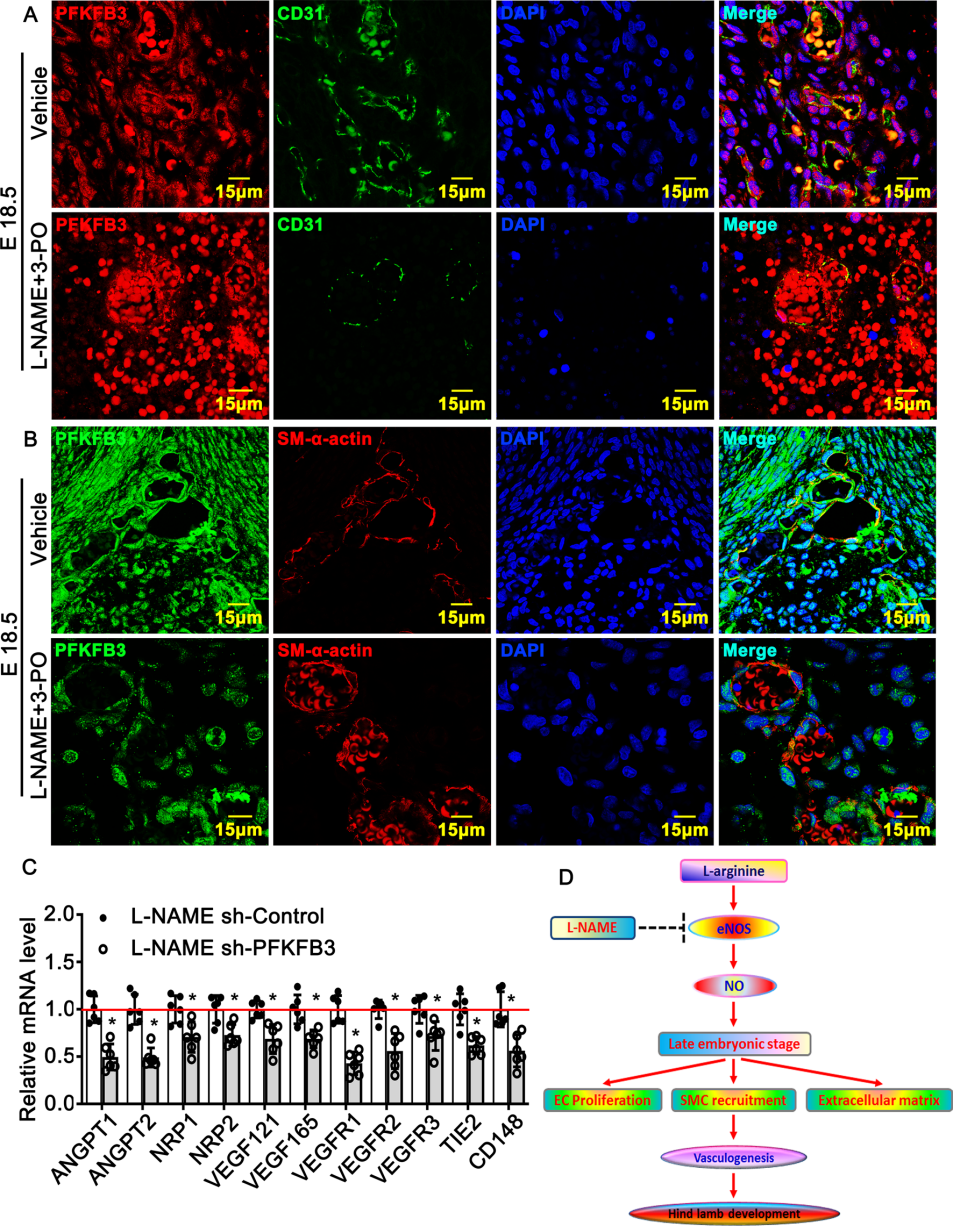
Suppression of NO production impairs Vasculogenesis through PFKFB3 mediated glycolytic signaling pathway. (**A**) NO synthesis inhibited by consecutive intraperitoneal injection of L-NAME, to pregnant rat starting from embryonic day 13.5 (E13.5). Following consecutive intraperitoneal injection 3-PO, PFKFB3 inhibitor, started at E16.5, and collected embryos at E18.5. Immunofluorescence staining against CD31 and PFKFB3 antibodies. (**B**) Immunofluorescence staining against SM α-actin and PFKFB3 antibodies at E18.5. Scale bar: 15μm. (**C**) HUVEC infected with sh-PFKFB3 or sh-Control to generate PFKFB3 knockdown endothelial cells. Treated those cells with L-NAME (1 μM) for 30 h and Real Time PCR analysis expression of angiogenetic genes (n = 6 independent experiments, paired *t*-test). The experiments repeated three time. Data presented as means ± SEM. P < 0.05. (**D**) Schematic diagram demonstrated l-arginine-eNOS-NO pathway plays a critical role in regulation rat embryonic hind limb development, which promotes angiogenetic remodeling through PFKFB3 mediated glycolytic signaling pathway.

We treated HUVEC with L-NAME and performed Real Time PCR to detect the expression of extracellular genes. Our data indicating that L-NAME treatment significant suppress CD31, GJC1, EFNB2 and Vcam1 expression (S Figure 6A). Similar results were exhibited in our in vitro studies using Gomori Methamine Silver Staining, which indicated that eNOS inhibition dramatically suppresses eticulin fiber and collagen deposition (S Figure 6B). However, L-NAME treatment did not alter the tubulogenesis gene expression, including RHOA F3 and EGFL7 (S Figure 6C). Taken together, L-NAME treatment was not critical for tubulogenesis, However, L-NAME treatment plays a pivotal role in regulating angiogenic remodeling.

### Blocking l-arginine/eNOS/NO pathway suppresses vascular wall PFKFB3 expression

Our previously study and other groups demonstrate that PFKFB3 mediated glycolysis signaling pathway is essential for angiogenesis^28,36^. However, whether glycolytic signaling pathway is crucial for rat late embryonic hind lamb Vasculogenesis which controlled by NO production is still large unknown. We treated HUVEC with L-NAME and performed real time PCR to screen the expression of glycolytic genes. Our data showed that L-NAME treatment significantly decreased glycolytic genes expression, such as PFKFB3, PGK1, PFKP, PDK1 and ALDOA (S Figure 7). Since PFKFB3 is the rate-limiting enzyme during glucose metabolism, we further validated whether L-NAME treatment can suppress the expression of PFKFB3 in vivo and in vitro. We treated HUVEC with different dose of L-NAME, including 0.5 μM, 1.0 μM and 5.0 μM. L-NAME dramatically suppresses the expression of PFKFB3 at both protein level and mRNA level (Fig. 5A–C). At E16.5, high expression level of PFKFB3 detected in nonucleated hematopoietic precursor cells resident in both control group and L-NAME treated group (data no shown). Whereas PFKFB3 expression was significantly decreased within the cells around tubular structure vessels at E17.5 in L-NAME treated group (S Figure 8). At E18.5, PFKFB3 ubiquitin expressed in rat embryonic hind limb. However, the expression level of PFKFB3 within vascular wall is much lower after L-NAME treatment, as well decreased IOD, vessels wall thickness and enlargement of vessel lumen (S Figure 9A,B). The similar results exhibited at E20.5 stage (S Figure 9C,D). Those data indicated that inhibiting production of NO from l-arginine by L-NAME leads to decrease the expression of PFKFB3 in vascular wall during rat late embryonic hind limb development.

### PFKFB3 mediated glycolytic signaling pathway is involved in l-arginine/eNOS/NO pathway in regulating rat late embryonic hind limb development

Glycolysis regulates EC rearrangement during vessel sprouting^40^. Nitric oxide increase the activity of 6-phosphofructo-1-kinase (PFK1), a master regulator of glycolysis, to regulate glycolysis^41,42^. PFKFBs catalyze the synthesis and degradation of fructose 2, 6-bishosphate (F2, 6 BP), a powerful activator of PFK1, the rate-limiting enzyme in glycolytic flux. Four isoforms of PFK-2 been identified in mammal cells, which encoded by the genes PFKFB1, PFKFB2, PFKFB3 and PFKFB4. PFK2 that encoded by PFKFB3 plays a key role in the regulation of endothelial glycolytic flux. However, whether Nitric Oxide promotes angiogenesis through upregulating the expression of PFKFB3 mediated glycolysis is largely unknown.

In order to explore this hypothesis, we first determined whether L-NAME treatment suppresses the expression of Vasculogenesis genes by real time PCR. Our results indicate that L-NAME dramatically inhibited ANGPT1, ANGPT2, VEGF165, VEGFR2 and CD148 (S Figure 10). We next sought to determine whether blocking l-arginine/eNOS/NO suppresses late embryonic hind limb vasculogenesis through PFKFB3 mediated glycolysis pathway. We performed immunofluorescence staining against endothelial marker gene CD31 and PFKFB3. At E18.5, high expression level of CD31 and PFKFB3 detected within endothelial cells in control groups. However, blocking l-arginine/eNOS/NO significant decreases CD31 expression, as well as PFKFB3 expression within endothelial cells (Fig. 5D). We also performed Immunofluorescence staining against smooth muscle marker gene SM a-action and PFKFB3. At E20.5, blocking l-arginine/eNOS/NO dramatically suppresses SM a-action and PFKFB3 expression within vascular wall (Fig. 5E). The data indicated that blocking l-arginine/eNOS/NO suppresses late embryonic hind limb vasculogenesis through PFKFB3 mediated glycolysis pathway.

### Inhibition of PFKFB3 mediated glycolytic signaling pathway exacerbates late embryonic hind limb developmental defects that caused by l-arginine/eNOS/NO pathway impairment

We reduced NO synthesis by consecutive intraperitoneal injection of L-NAME, to pregnant rat starting from embryonic day 13.5 (E13.5). Following consecutive intraperitoneal injection of 3-PO, PFKFB3 inhibitor, stared at E16.5 and collected embryos at E18.5^43^. We performed immunofluorescence staining against endothelial marker gene CD31 and PFKFB3. After inhibition both NO and PFKFB3, the expression of CD31 was dramatically decreases (Fig. 6A). The expression of SM a-actin was significant decrease (Fig. 6B). Adenovirus encoding PFKFB3 was used to knockdown PFKFB3 in HUVEC. We check PFKFB3 knockdown efficiency by Real Time PCR and Western blot (S Figure 11). We next treated HUVEC with L-NAME after infected with sh-Control or sh-PFKFB3. Our Real Time PCR data demonstrated that knockdown of PFKFB3 in L-NAME treated HUVEC dramatically suppressed angiogenetic genes, including Angiopoietin, NRP1, VEGF121, VEGF165, VEGFR2, Tie2 as well as CD148 (Fig. 6C). Tubulogenesis was inhibited which characterized by attenuated expression of Rhoa and F3 (S Figure 12A). The remodeling of new vessels was dramatically suppressed which associated with inhibition extracellular matrix deposition and Notch signaling pathway (S Figure 12B; S Figure 13A,B). Our data indicate PFKFB3 is critical for l-arginine-eNOS-NO pathway in regulating new vessels remodeling.

Overall, the results indicated that l-arginine-eNOS-NO pathway plays a critical role in regulation rat embryonic hind limb development, which promotes angiogenetic remodeling through PFKFB3-mediated glycolytic signaling pathway (Fig. 6D).

## Discussion

We provide the first evidence in this study demonstrating that l-arginine-eNOS-NO pathway is critical for rat hind limb late embryonic development through PFKFB3 mediated angiogenesis. We consecutive treated pregnant rat with L-NAME to inhibit eNOS by intraperitoneal injection, and more than 20% of the embryos exhibited severe hind limb developmental defects (Fig. 1A,B). H&E staining and immunohistochemistry staining indicate that l-arginine-eNOS-NO had no effect on the angiogenetic tubulogenesis. This was consistent with the results from in vitro studies on HUVEC. L-NAME treatment did not suppress HUVEC proliferation and migration. However, l-arginine-eNOS-NO pathway plays a pivotal role in regulating angiogenic remodeling, which characterized by impairing endothelial cells maturation, smooth muscle cells maturation and recruitment, as well as inhibition extracellular matrix deposition. The defection of new vessels remodeling leads to vascular unstabilization and vessel permeability, which resulting in vascular blood cell leakage, eventual leads to rat hind limb late embryonic development defects.

### Blocking l-arginine/eNOS/NO pathway causes late embryonic hind limb developmental defects

L-NAME is a Non-selective NO synthase inhibitor. L-NAME treatment suppresses NO production in multiple organs. There exists three distinct NOS isoforms: nNOS, iNOS, eNOS^6^. We determined which isoform dominant expressed in endothelial cells. The expression level of eNOS is much higher than that of iNOS or nNOS. L-NAME treatment dramatically inhibited eNOS expression, while not influenced the expression of iNOS and eNOS. Most embryos exhibited severe hind limb defects. We also observed that no more than 5% of the embryos exhibited fore hind limb defects and less than 1% of the embryos shown cerebral defects. NO was reported to regulate preimplantation embryo development, especially for development of two cells embryos to four cells stage^44,45^. eNOS knockout mice has been generated to observe early embryonic development^46^. However, the roles of NO that regulated by NOS on late embryonic development is largely unknown. In this study, we suppressed eNOS by L-NAME to determine late embryonic hind limb development.

### Blocking l-arginine/eNOS/NO pathway caused late embryonic hind limb through PFKFB3 mediated glycolysis

The function role of Nitric Oxide in cardiovascular system reported to determine basal vascular tone, prevents platelet activation, limits leukocytes adhesion to endothelium, and regulates myocardial contractility^47^. Nitric Oxide signaling is critical for endothelial differentiation of embryonic stem cell^48^. At late embryonic stage, the development of circulatory system is mostly dependent on endothelial cells through sprouting angiogenesis^36^.

The vascular tubulogenesis is strictly dependent proliferation of stalk cells and the migration of tip cells. We reduced NO synthesis by consecutive intraperitoneal injection of L-NAME, an unselected NOS inhibitor, to pregnant rat starting from embryonic day 13.5. The defection of hind limb identified at E18.5, number of osteocyte decreased within fingers. More severe phenotypes exhibited at E19.5, almost all bone tissues were disappeared, and infiltration of blood cell observed. Tissue necrosis happened at E20.5, which characterized cell fragments, nucleus without cytoplasm, and dissolved extracellular matrix. Blocking l-arginine/eNOS/NO pathway by L-NAME treatment impairs angiogenetic remodeling characterized by inhibition maturation of endothelial cells and smooth muscle cells, as well as suppressing extracellular matrix deposition, which contribute to stabilization of new vessels.

In this study, we identified a novel mechanism whereby l-arginine-eNOS-NO pathway in control rat late embryonic hind limb development which characterized by impairing angiogenesis through PFKFB3 mediated glycolysis. Our data indict that L-NAME treatment dramatically suppresses PFKFB3 expression in hematopoietic progenitor cell, tubulogenetic endothelial cells and smooth muscle cells. Knockdown of PFKFB3 by infection HUVEC with adenoviral PFKFB3 resulting in dramatically inhibition expression of angiogenetic genes, as well as tubulogenesis and extracellular matrix related genes. This treatment drastically suppresses Notch signaling pathway, which is essential for regulating angiogenic remodeling (S Figure 11). We failed to mimick hind limb developmental phonotype by using 3-PO, a PFKFB3 inhibitor. The likely explanation could be that ubiquitin expression of PFKFB3 has multiple functions for multiple organs. We did observe severe hemorrhage in addition to hind limb area.

## Conclusions

Taken together, our data in this study demonstrate that l-arginine-eNOS-NO pathway is important for rat hind limb development during late postembryonic stage. This could be both a useful animal model and a promising therapeutic target for congenital defects of developmental hind limbs.

## Supplementary information

Supplementary Information.

Supplementary Figures.

## Data Availability

The authors state that all relevant data are available within the article and the online Supplementary material or are available from the corresponding authors upon reasonable request.
