# Publisher Correction: Inhibition of eNOS by L-NAME resulting in rat hind limb developmental defects through PFKFB3 mediated angiogenetic pathway

**DOI:** 10.1038/s41598-021-82924-8

**Published:** 2021-02-16

**Authors:** Ziqiang Wu, Huan Yao, Huan Xu, Yang Wang, Wangming Hu, Guanhua Lou, Lingling Zhang, Cong Huang, Cen Jiang, Shiyi Zhou, Yaping Shi, Xiongbing Chen, Lan Yang, Yiming Xu, Yong Wang

**Affiliations:** 1 Chengdu University of Traditional Chinese Medicine, College of Basic Medicine, Chengdu, Sichuan China; 2 Chengdu University of Traditional Chinese Medicine, College Pharmacy, Chengdu, China; 3 School of Basic Medical Sciences, Guangzhou Medical University, Guangzhou, China

Correction to: *Scientific Reports* https://doi.org/10.1038/s41598-020-74011-1, published online 07 October 2020

In the original version of this Article, Ziqiang Wu, Huan Yao and Huan Xu were omitted as equally contributing authors. This has now been corrected in the PDF and HTML versions of the Article, and in the accompanying Supplementary Information files.

