# Supplementary material for: Inhibition of eNOS by L-NAME resulting in rat hind limb developmental defects through PFKFB3 mediated angiogenetic pathway
Source: Sci Rep. 2020 Oct 7;10:16754. doi: 10.1038/s41598-020-74011-1 (PMC7541470; doi:10.1038/s41598-020-74011-1)
Supplement: Supplementary file 1 — Supplementary Information. [file 41598_2020_74011_MOESM1_ESM.doc]

**Supplementary data:**

**Inhibition of eNOS by L-NAME resulting in rat hind limb developmental defects through PFKFB3 mediated angiogenetic pathway**

Ziqiang Wu ^1, 2 †^, Huan Yao ^1†^, Huan Xu ^1†^, Yang Wang ^1^, Wangming Hu ^1^, Guanhua Lou ^2^, Lingling Zhang ^1^, Cong Huang ^1^, Cen Jiang ^1^, Shiyi Zhou ^1^, Yaping Shi ^1^, Xiongbing Chen ^1^, Lan Yang ^1^, Yiming Xu ^3^, Yong Wang ^1*^

^1^ Chengdu University of Traditional Chinese Medicine, College of Basic Medicine, Chengdu, China.

^2^ Chengdu University of Traditional Chinese Medicine, College Pharmacy, Chengdu, China.

^3^ Guangzhou Medical University, School of Basic Medical Sciences, Guangzhou, China.

^†^ Ziqiang Wu, Huan Yao and Huan Xu contributed equally to this work

* Corresponding author: Yong Wang, PhD.

Basic Medical College,

Cheng Du University of Traditional Chinese Medicine,

Chengdu, Sichuan China.

Email: [yongwang1008@hotmail.com](mailto:yongwang1008@hotmail.com)

wangyong@cdutcm.edu.cn

**Supplementary Figures:**

S Figure 1. Suppression of No production impairs hind limb angiogenesis. A. Representative Images of HE staining from E16 to exhibit the vessel structure within hind limb, the whole images exhibited in left and corresponding areas exhibited in middle and right. Quantification analysis of endothelial cell numbers for each vessel displayed in B (n=8 mice in per group). C. Representative Images of HE staining from E17 and analysis of endothelial cell numbers and thickness for each vessel exhibited in D (n=9 mice in per group) and E (n=7 mice in per group). Representative Images of HE staining from E19 and analysis of endothelial cell numbers and thickness for each vessel exhibited in F, G (n=8 mice in per group) and H (n=6 mice in per group). Data presented as means ± SEM, *p˂0.05. Scale bar: 60μm.

S Figure 2. eNOS is the major isoform of NOS in endothelial cells. A. Total RNA extracted from HUVEC and Real Time PCR analysis the mRNA level of eNOS, iNOS and nNOS. eNOS is the major isoform of NOS in endothelial cells (n=6 independent experiments). B. HUVEC treated with L-NAME treatment (1μM) or vehicle for 30 hours, Total RNA extracted and Real Time PCR analysis the mRNA level of eNOS, iNOS and nNOS (n=6 independent experiments). L-NAME treatment significant suppresses eNOS expression in endothelial cells. Data presented as means ± SEM, *p˂0.05.

S Figure 3. Suppression of No production suppresses vascular endothelial cells development and maturation. A. IHC staining against CD31 antibody to visualize the vascular endothelial cells development at E16. Relative expression of CD31 according to IOD and vessel numbers quantified in B (n=4 mice per group) and C (n=4 mice per group). IHC staining against CD31 antibody at E17, and analysis data exhibited in D, E (n=4 mice per group), F (n=4 mice per group). G. IHC staining against CD31 antibody at E20 and analysis data shown in H (n=4 mice per group) and I (n=4 mice per group). Data represented as means ± SEM, *p˂0.05. Scale bar: 60μm.

S Figure 4. Suppression of No production impairs smooth muscle cell development and recruitment. A. IHC staining against SM α-actin to visualize the vascular smooth muscle cell development at E17. B. Relative expression of SM α-actin was quantified which based on IOD (n=6 mice per group). IHC staining against smooth muscle SM α-actin at E19 and SM α-actin relative protein level shown in C and D (n=4 mice per group). Data represented as means ± SEM, *p˂0.05. Scale bar: 60μm.

S Figure 5. Suppression of No production impairs smooth muscle cell development and recruitment. A. IHC staining against MHC to visualize the vascular smooth muscle cell development at E18.5. B. Relative expression of MHC quantified which based on IOD (n=4 mice per group). IHC staining against smooth muscle MHC at E19 relative protein level shown in C and D (n=4 mice per group). Data represented as means ± SEM, *p˂0.05. Scale bar: 60μm.

S Figure 6. Suppression of No production impairs endothelial extracellular matrix deposition. A. HUVEC treated with L-NAME (1μM) for 30 hours and Real Time PCR analysis expression of extracellular matrix genes (n=6 independent experiments). B. Gomori Methamine Silver Staining to show eticulin fiber and collagen deposition at E17 embryos from L-NAME or Control groups. C. The expression of vascular tubulegenetic genes in HUVEC after L-NAME treatment (n=6 independent experiments). Data represented as means ± SEM, *p˂0.05.

S Figure 7. Suppression of No production suppresses endothelial glycolytic genes expression. HUVEC treated with L-NAME (1μM) for 30 hours and Real Time PCR analysis expression of glycolytic genes (n=6 independent experiments). Data represented as means ± SEM, *p˂0.05.

S Figure 8. Immunohistochemistry staining against PFKFB3 to determine the expression of PFKFB3 in vascular endothelial cell at E17.5.

S Figure 9. Suppression of No production impairs PFKFB3 expression. A. IHC staining against PFKFB3 to visualize the vascular smooth muscle cell development at E18.5. B. Relative expression of PFKFB3 was quantified which based on IOD (n=6 mice per group). IHC staining against PFKFB3 at E20.5 and PFKFB3 relative protein level shown in C and D (n=4 mice per group). Data represented as means ± SEM, *p˂0.05. Scale bar: 60μm.

S Figure 10. Suppression of No production suppresses endothelial glycolytic genes expression. HUVEC treated with L-NAME (1μM) for 30 hours and Real Time PCR analysis expression of glycolytic genes (n=6 independent experiments). Data represented as means ± SEM, *p˂0.05.

S Figure 11. Validating PFKFB3 knockdown efficiency in HUVEC. HUVEC infected with sh-Control or sh-PFKFB3, Real Time PCR and western blot performed to validate mRNA (A) (n=5 independent experiments) and protein (B) knockdown efficiency. Data represented as means ± SEM, *p˂0.05.

S Figure 12. PFKFB3 mediated glycolysis pathway is involved in L-arginine/eNOS/NO pathway in regulating angiogenesis. A. HUVEC treated with L-NAME (1μM) after infected with sh-PFKFB3 or sh-Control, and Real Time PCR analysis detect tubulogenesis genes, the expression of extracellular matrix genes exhibited in B. Data represented as means ± SEM, *p˂0.05.

S Figure 13. L-arginine/eNOS/NO is associated Notch signaling pathway. A. HUVEC treated with L-NAME (1μM), and Real Time PCR performed to detect the expression of Notch signaling pathway targets. B. HUVEC treated with L-NAME (1μM) after infected with sh-PFKFB3, and Real Time PCR performed to detect the expression of Notch signaling pathway targets.
