# Supplementary material for: Inhibition of eNOS by L-NAME resulting in rat hind limb developmental defects through PFKFB3 mediated angiogenetic pathway
Source: Sci Rep. 2020 Oct 7;10:16754. doi: 10.1038/s41598-020-74011-1 (PMC7541470; doi:10.1038/s41598-020-74011-1)
Supplement: Supplementary file 2 — Supplementary Figures. [file 41598_2020_74011_MOESM2_ESM.pdf]

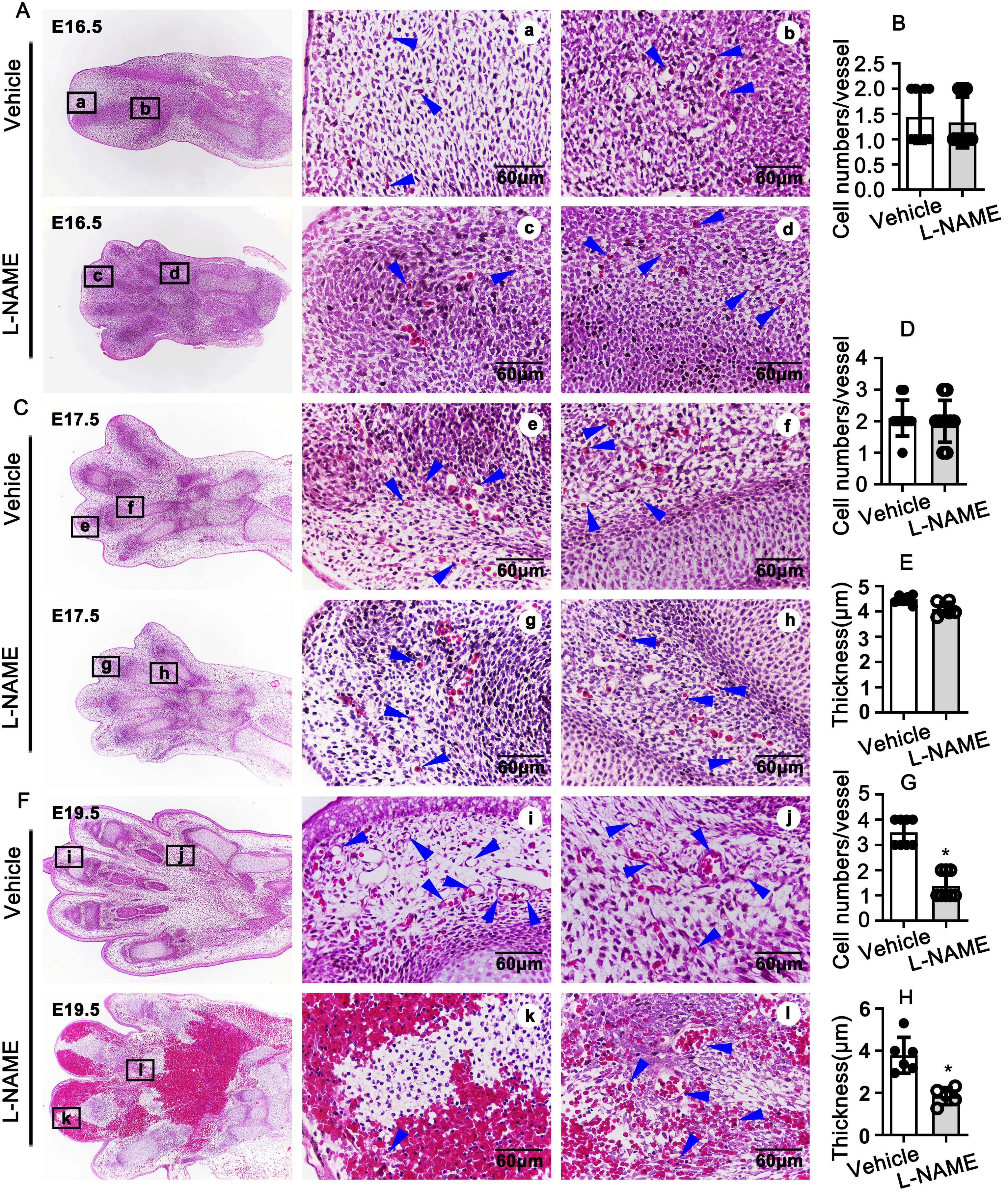

A
E16.5
Vehicle
a
b
60μm
B
Cell numbers/vessel
2.5
2.0
1.5
1.0
0.5
0.0
Vehicle
L-NAME
E16.5
L-NAME
c
d
D
4
3
2
1
0
C
E17.5
e
f
E
Thickness(μm)
5
E17.5
g
h
G
*
F
E19.5
i
j
E19.5
k
l
H
6

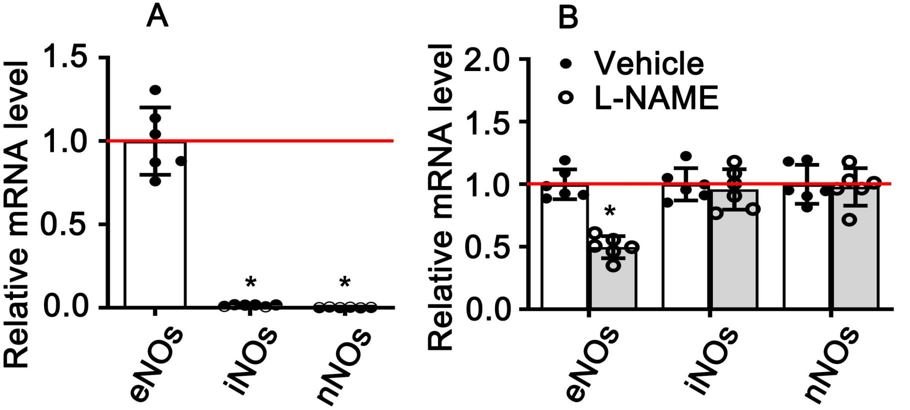

A
Relative mRNA level
1.5
1.0
0.5
0.0
*
*
eNOs
iNOs
nNOs
B
Relative mRNA level
2.0
1.5
1.0
0.5
0.0
Vehicle
L-NAME
*
eNOs
iNOs
nNOs

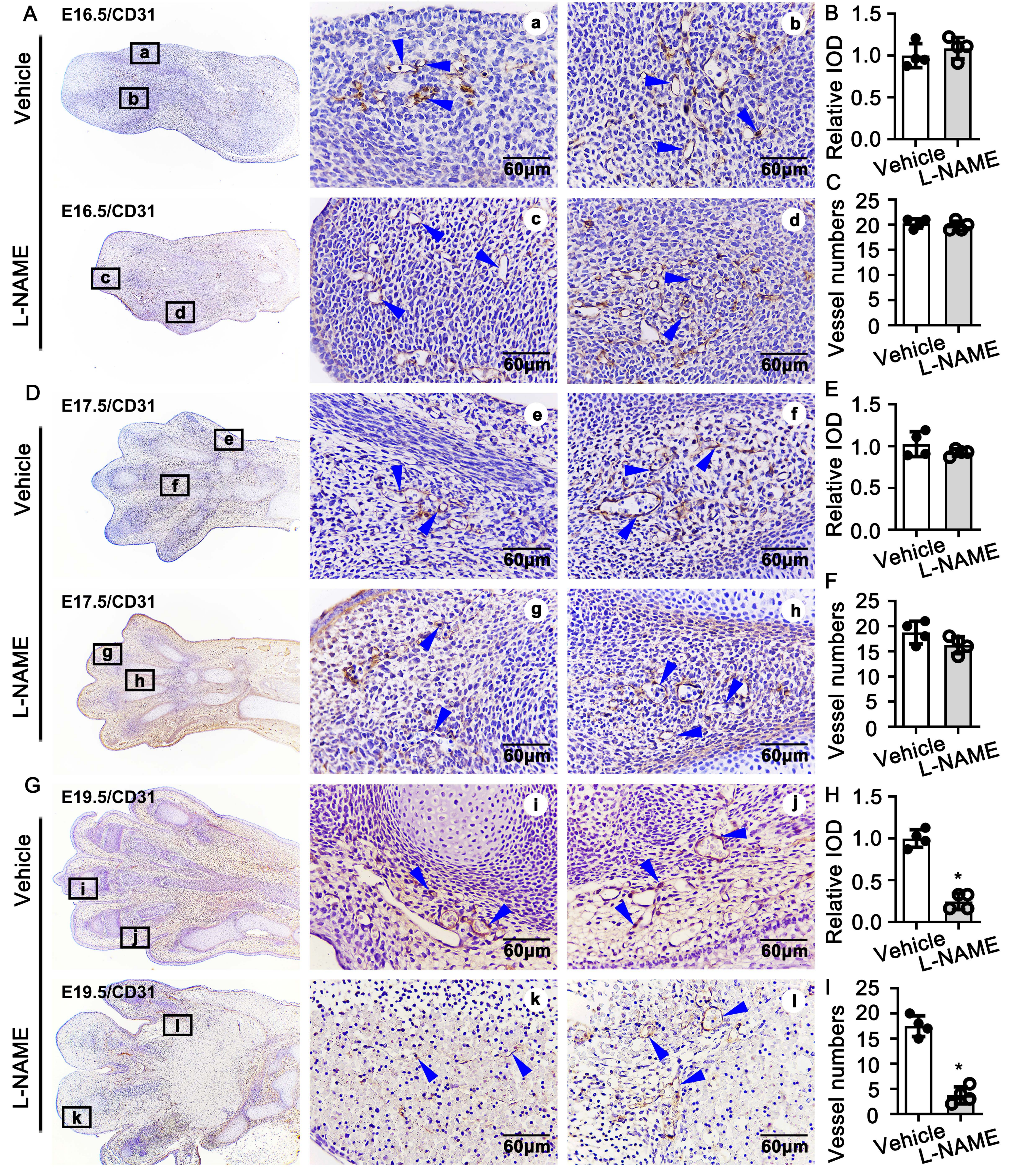

A
E16.5/CD31
Vehicle
L-NAME
a
b
c
d
60μm
B
Relative IOD
1.5
1.0
0.5
0.0
Vehicle
L-NAME
C
Vessel numbers
25
20
15
10
5
0
D
E17.5/CD31
e
f
g
h
E
F
G
E19.5/CD31
i
j
k
l
H
I
*

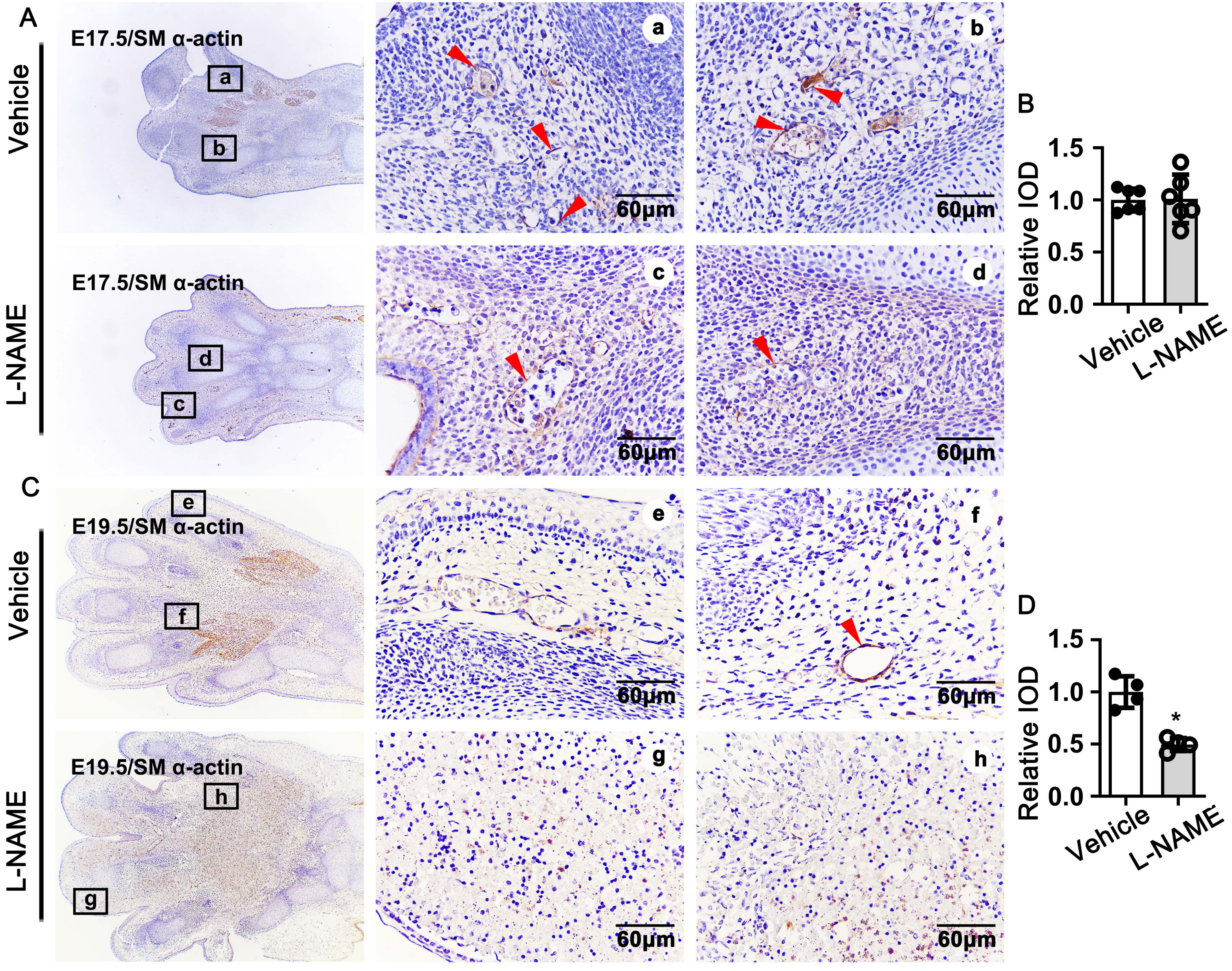

A
E17.5/SM α-actin
Vehicle
a
b
60μm
60μm
E17.5/SM α-actin
L-NAME
c
d
60μm
60μm
B
Relative IOD
1.5
1.0
0.5
0.0
Vehicle
L-NAME
C
E19.5/SM α-actin
Vehicle
e
f
60μm
60μm
E19.5/SM α-actin
L-NAME
g
h
60μm
60μm
D
Relative IOD
1.5
1.0
0.5
0.0
*
Vehicle
L-NAME

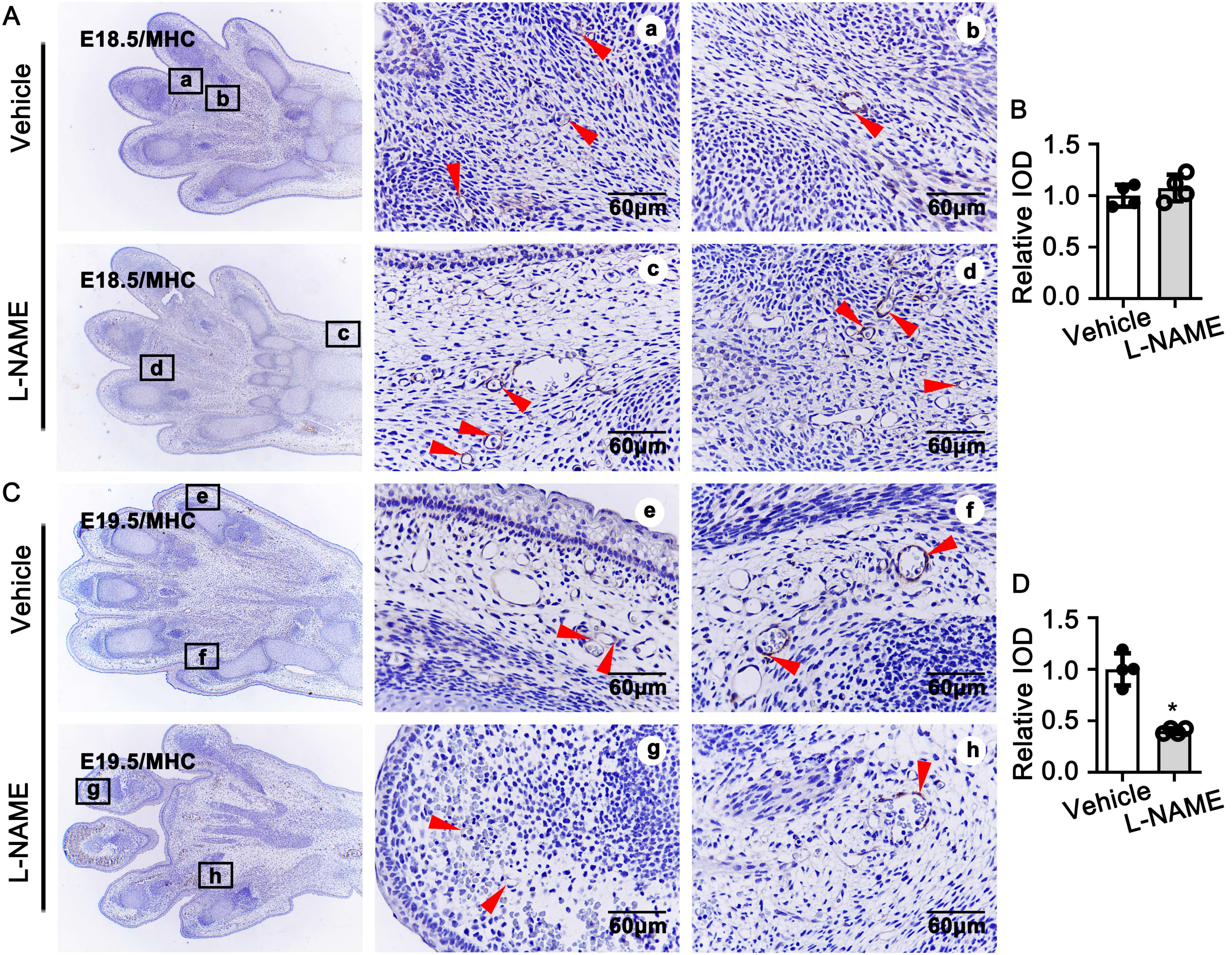
A
E18.5/MHC
Vehicle
a
b
60μm
60μm
B
1.5
1.0
0.5
0.0
Relative IOD
Vehicle
L-NAME
E18.5/MHC
L-NAME
c
d
60μm
60μm
C
E19.5/MHC
Vehicle
e
f
60μm
60μm
D
1.5
1.0
0.5
0.0
Relative IOD
*
Vehicle
L-NAME
E19.5/MHC
L-NAME
g
h
60μm
60μm

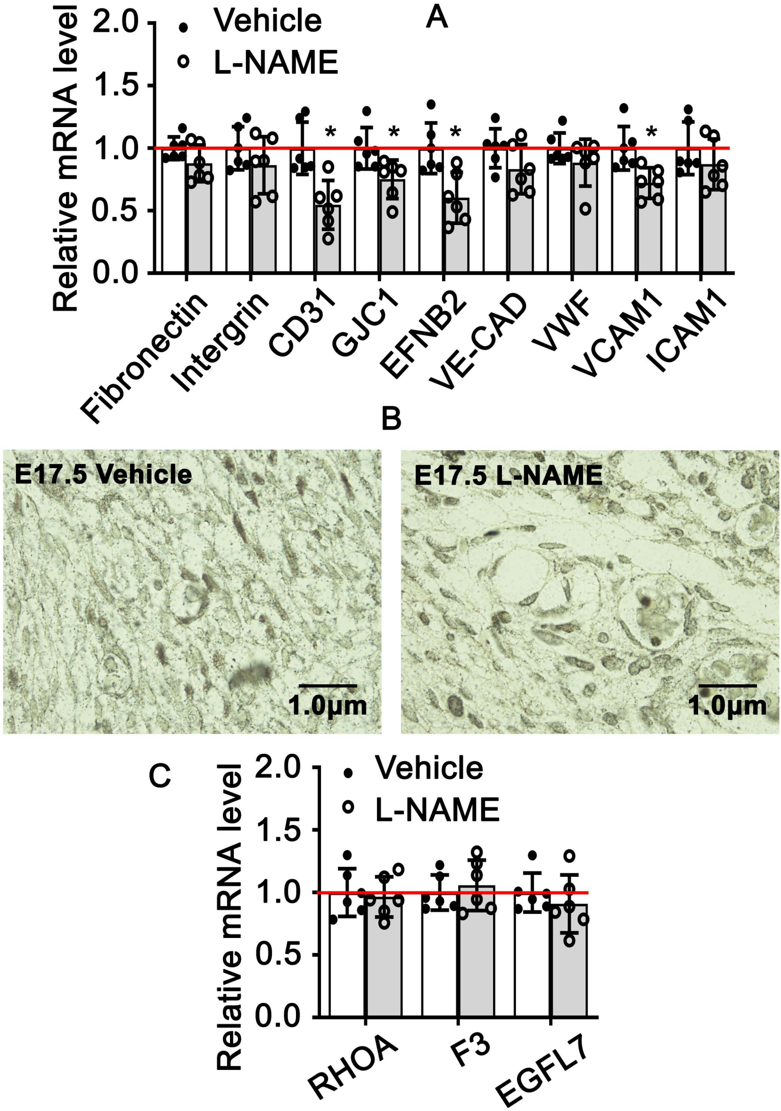
A
Relative mRNA level
2.0
1.5
1.0
0.5
0.0
Vehicle
L-NAME
*
Fibronectin
Intergrin
CD31
GJC1
EFNB2
VE-CAD
VWF
VCAM1
ICAM1
B
E17.5 Vehicle
E17.5 L-NAME
1.0μm
C
RHOA
F3
EGFL7

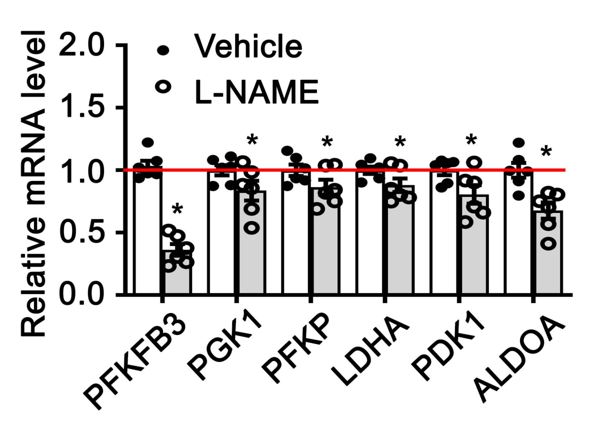

Relative mRNA level
2.0
1.5
1.0
0.5
0.0
Vehicle
L-NAME
*
*
*
*
*
*
PFKFB3
PGK1
PFKP
LDHA
PDK1
ALDOA

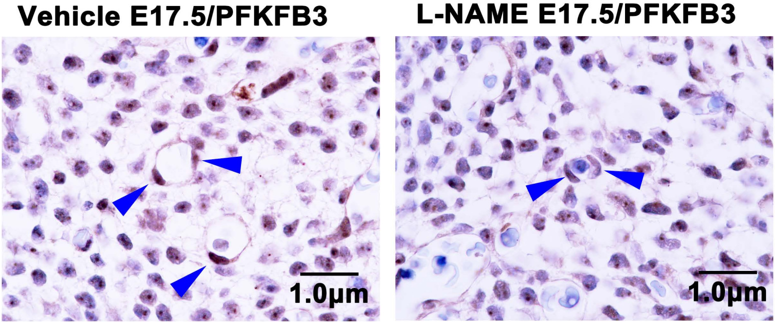
Vehicle E17.5/PFKFB3
L-NAME E17.5/PFKFB3
1.0μm
1.0μm

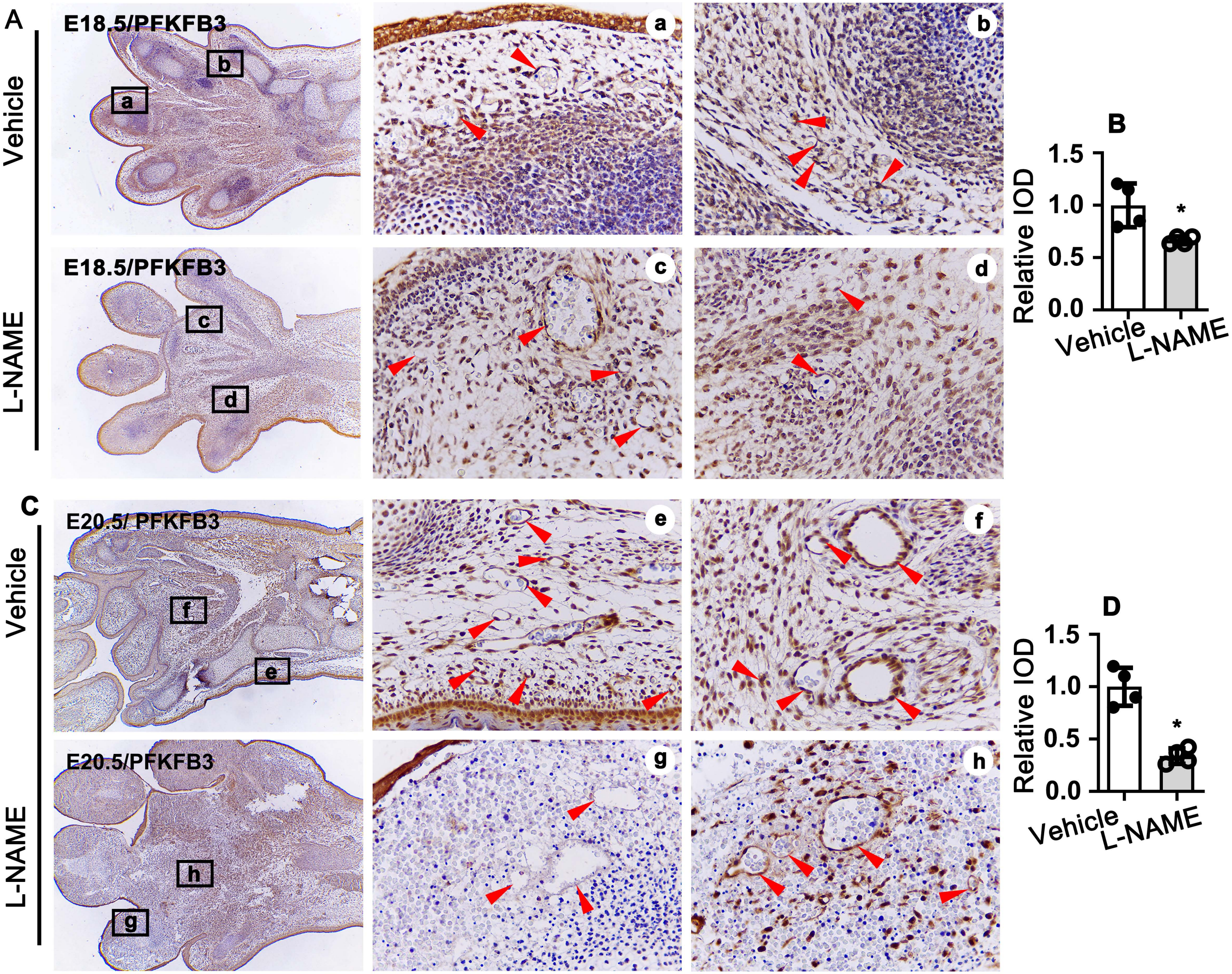

A
E18.5/PFKFB3
Vehicle
L-NAME
a
b
c
d
B
Relative IOD
1.5
1.0
0.5
0.0
Vehicle
L-NAME
*
C
E20.5/ PFKFB3
E20.5/PFKFB3
e
f
g
h
D

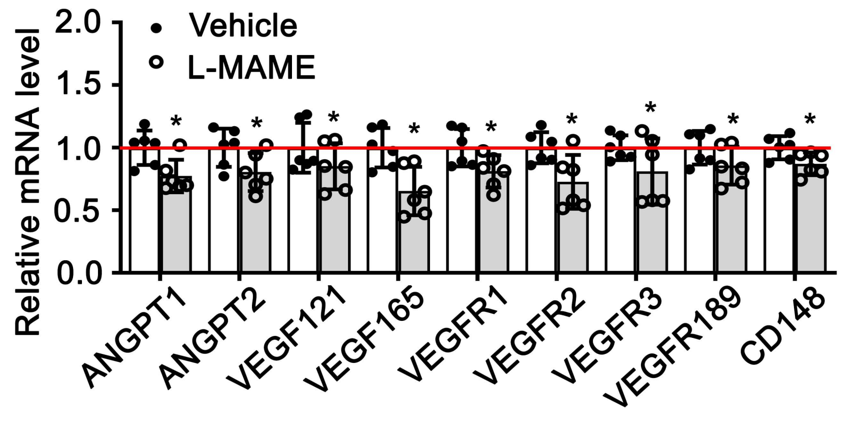

Vehicle
L-MAME
Relative mRNA level
2.0
1.5
1.0
0.5
0.0
*
ANGPT1
ANGPT2
VEGF121
VEGF165
VEGFR1
VEGFR2
VEGFR3
VEGFR189
CD148

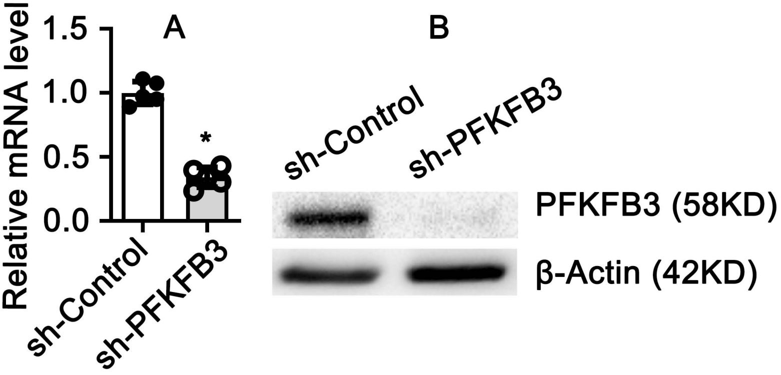
A
B
Relative mRNA level
1.5
1.0
0.5
0.0
*
sh-Control
sh-PFKFB3
sh-Control
sh-PFKFB3
PFKFB3 (58KD)
β-Actin (42KD)

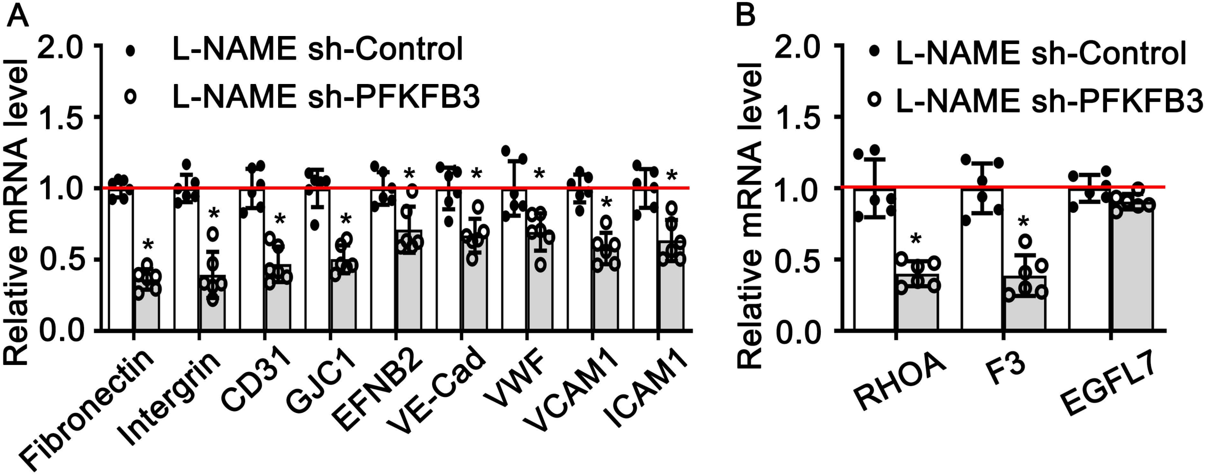
A
B
Relative mRNA level
2.0
1.5
1.0
0.5
0.0
L-NAME sh-Control
L-NAME sh-PFKFB3
Fibronectin
Intergrin
CD31
GJC1
EFNB2
VE-Cad
VWF
VCAM1
ICAM1
RHOA
F3
EGFL7
*

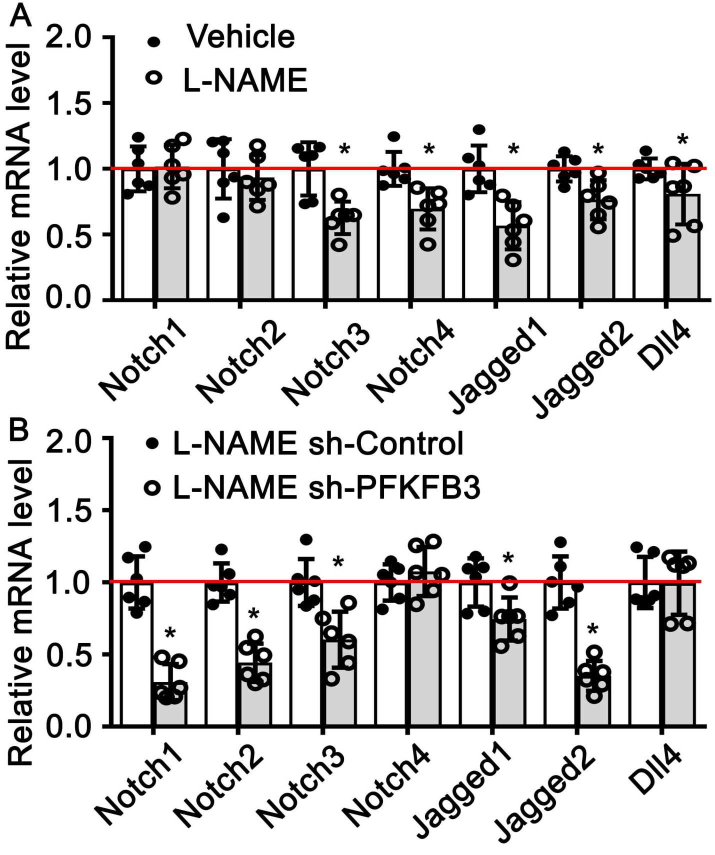
A
Vehicle
L-NAME
Relative mRNA level
2.0
1.5
1.0
0.5
0.0
*
*
*
*
*
Notch1
Notch2
Notch3
Notch4
Jagged1
Jagged2
Dll4
B
L-NAME sh-Control
L-NAME sh-PFKFB3
Relative mRNA level
2.0
1.5
1.0
0.5
0.0
*
*
*
*
*
Notch1
Notch2
Notch3
Notch4
Jagged1
Jagged2
Dll4
